# Postoperative peri-axillary seroma following axillary artery cannulation for surgical treatment of acute type A aortic dissection

**DOI:** 10.1186/1749-8090-5-43

**Published:** 2010-05-25

**Authors:** Efstratios E Apostolakis, Nikolaos G Baikoussis, Konstantinos Katsanos, Menelaos Karanikolas

**Affiliations:** 1 Cardiothoracic Surgery Department, University of Patras, School of Medicine, Patras, Greece; 2 Department of Interventional Radiology, University of Patras, School of Medicine, Patras, Greece; 3 Department of Anaesthesiology and Intensive Care Medicine, University of Patras School of Medicine, Patras, Greece

## Abstract

The arterial cannulation site for optimal tissue perfusion and cerebral protection during cardiopulmonary bypass (CPB) for surgical treatment of acute type A aortic dissection remains controversial. Right axillary artery cannulation confers significant advantages, because it provides antegrade arterial perfusion during cardiopulmonary bypass, and allows continuous antegrade cerebral perfusion during hypothermic circulatory arrest, thereby minimizing global cerebral ischemia. However, right axillary artery cannulation has been associated with serious complications, including problems with systemic perfusion during cardiopulmonary bypass, problems with postoperative patency of the artery due to stenosis, thrombosis or dissection, and brachial plexus injury. We herein present the case of a 36-year-old Caucasian man with known Marfan syndrome and acute type A aortic dissection, who had direct right axillary artery cannulation for surgery of the ascending aorta. Postoperatively, the patient developed an axillary perigraft seroma. As this complication has, not, to our knowledge, been reported before in cardiothoracic surgery, we describe this unusual complication and discuss conservative and surgical treatment options.

## Introduction

The arterial cannulation site for optimal tissue perfusion and cerebral protection during cardiopulmonary bypass (CPB) for surgical treatment of acute type A aortic dissection remains controversial [1-3]. Avoidance of femoral artery cannulation may reduce the risk of retrograde embolic events from atheromatous debris in the thoracic and abdominal aorta, but direct ascending aorta cannulation can be complicated by the presence of thrombus or atheromatous debris [4,5]. Right axillary artery cannulation provides antegrade arterial perfusion during CPB and allows continuous antegrade cerebral perfusion during hypothermic circulatory arrest, thereby minimizing global cerebral ischemia [3,4]. However, right axillary artery cannulation has been associated with serious complications, including malperfusion problems during CPB, compromised postoperative patency of the axillary artery (due to stenosis, thrombosis or dissection) and brachial plexus injury[6,7]. Perigraft seroma is a rare complication in vascular surgery and, to our knowledge, has not been reported after axillary artery cannulation. We herein describe the case of a 36 year old man with Marfan syndrome and acute aortic dissection, who had right axillary artery cannulation for aortic root and ascending aorta replacement, and postoperatively developed a seroma in the right suclavian area.

## Case presentation

**Figure 1:**
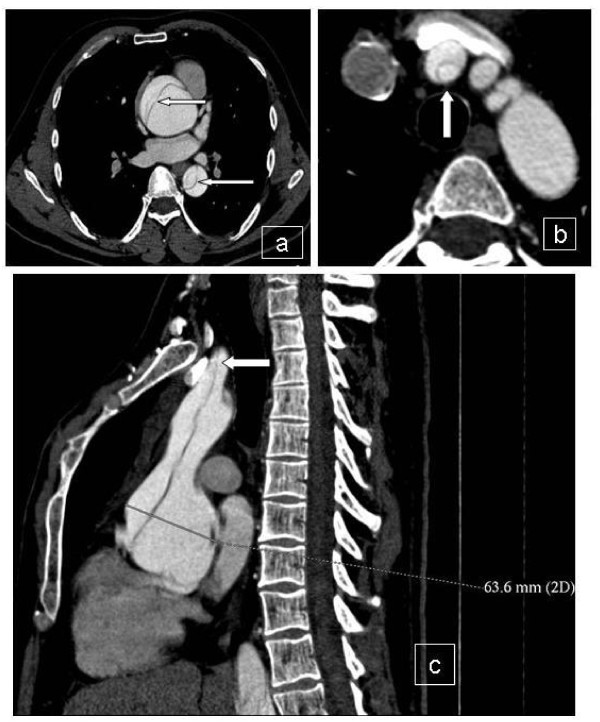
**CT scan with contrast reveals ascending aorta dilatation with intimal flap (arrows) in the ascending and descending aorta (a)**. Innominate artery dissection (b) and reconstructed image showing aortic root dilatation, together with aorta and innominate artery dissection (c).

**Figure 2:**
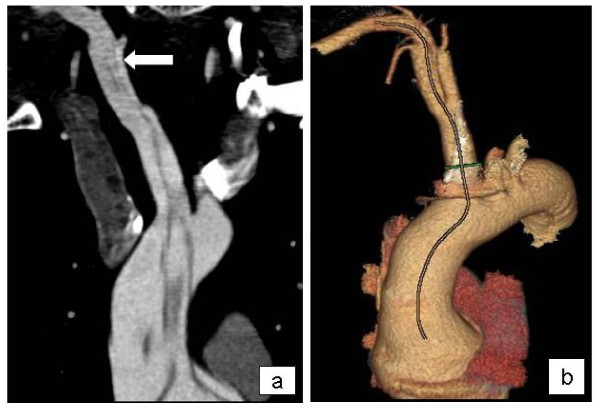
**Contrast-enhanced CT scan showing the intimal flap due to dissection from the aortic root to the ascending aorta, innominate artery and subclavian artery (a)**. Enhanced reconstructed CT scan image showing the path of dissection (b).

**Figure 3:**
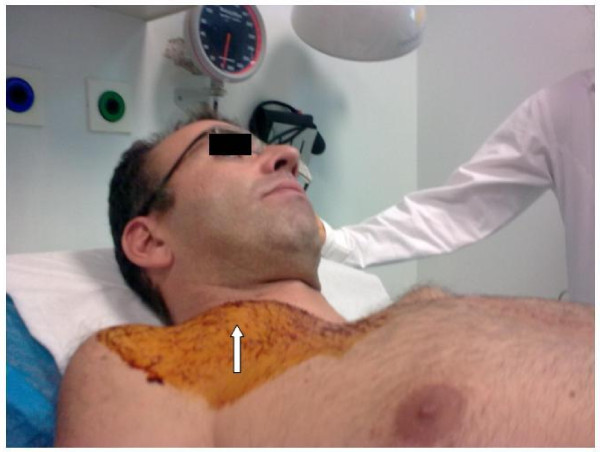
**Local, non-pulsatile swelling in the subclavian area (arrow) indicating a subcutaneous collection**.

**Figure 4:**
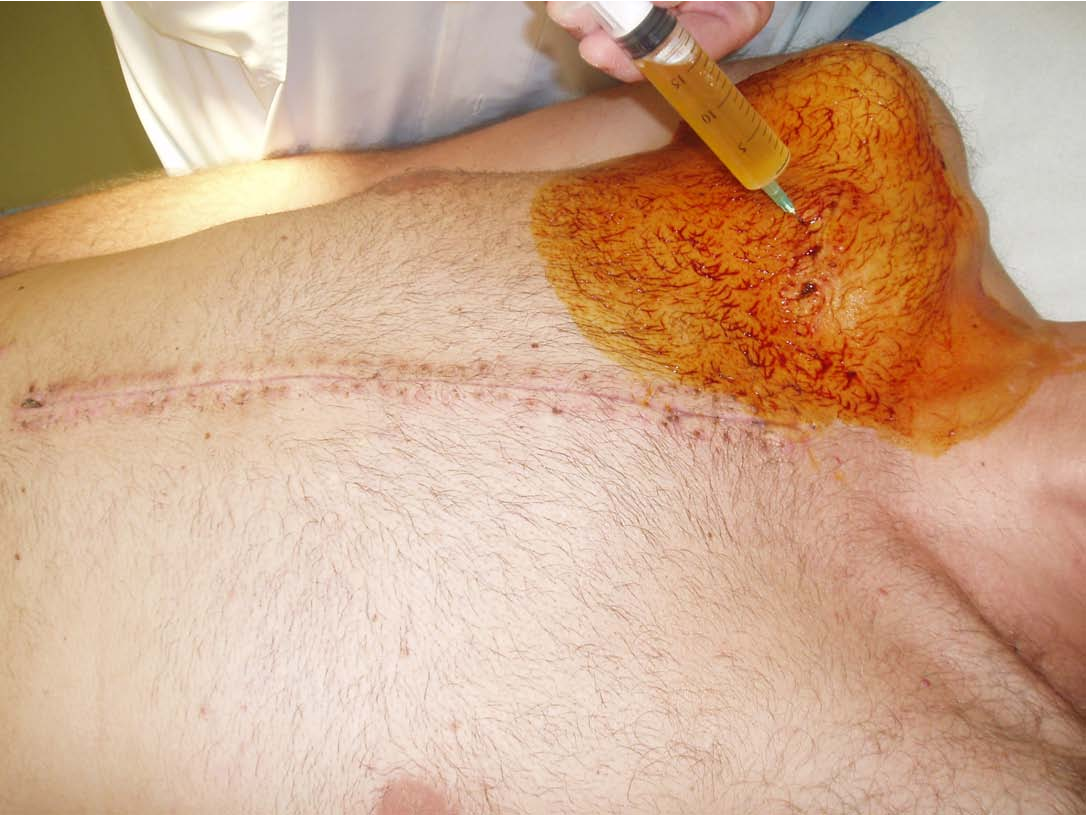
**Needle aspiration revealed serous, yellow fluid**.

A 36 year-old Caucasian man with Marfan syndrome was emergently admitted to our hospital with diagnosis of acute type A aortic dissection. Transthoracic echocardiography and computed tomography revealed aortic valve regurgitation and aortic dissection extending from the root of the aorta to the iliac arteries. The dissection extended into the arch vessels, involving mainly the innominate and axillary artery (figure 1, 2). The patient underwent the Bentall procedure under CPB instituted through direct right axillary artery cannulation, without interposition of an anastomotic graft. We did not use total hypothermic circulatory arrest; instead, continuous antegrade cerebral perfusion was achieved through cannulation of the right axillary artery, with the innominate artery clamped during arch reconstruction, using the "open distal anastomosis" technique. At the end of the operation, the subclavian artery cannulation site was repaired using a synthetic patch (Gore-tex Acuseal Cardiovascular patch, Gore & Associates, Flagstaff, Arizona 86004, USA). Initially we did not observe brachial plexus injury, bleeding, infection, vessel stenosis or any other complication related to axillary artery cannulation. However, local swelling was noted in the right subclavian area a week later, (figure 3). Needle aspiration revealed 50 ml of clear yellow transudate (figure 4), and laboratory analysis was negative for chylous collection (no chylomicrons, cholesterol/triglycerides >1). Total protein concentration of the liquid was 3.7 gm/dL, cholesterol 51 mg/dL, triglycerides 14 mg/dL and LDH 174 U/L. As swelling recurred after fluid aspiration, the patient required repeated needle aspiration every week for eight weeks. Three months after the operation, the seroma had disappeared, and did not recur. At his last follow-up six months after the operation, the patient was doing remarkably well: he had completely recovered from surgery had returned to his previous normal life, and swelling had completely disappeared.

## Discussion

Local complications after axillary artery cannulation can occur either intraoperatively (mostly technical problems, such as arterial injury with bleeding or malperfusion) [1,6-9], or postoperatively (mostly neurologic complications related to brachial plexus injury) [1,4,10]. Compared to the common femoral artery, the axillary artery is located deeper in tissues, in the vicinity of the brachial plexus, and this deep position likely contributes to higher incidence of cannulation-related complications [6,10]. Strauch et al [1] reported 14 complications among 284 patients who had axillary artery cannulation for surgery of the proximal aorta, with brachial plexus injury being the most common complication. Axillary perigraft seroma was not listed as a complication in this or any other relevant published clinical study. From the pathophysiological point of view, perigraft seromas consist of a clear, sterile fluid collection confined within the non-secreting fibrous pseudomembrane surrounding the implanted graft, and occur in 1.7% to 2.3% of all graft implantations in vascular surgery [11]. Knitted Dacron and polytetrafluorethylene are the materials most commonly implicated, with a higher percentage involving knitted Dacron grafts [11,12]. During the normal incorporation process of an implanted vascular graft, firmly adherent fibrous tissue and healthy wall matrix lining cover the graft by the 6th postoperative week [13], while seromas develop when the surrounding connective tissue fails to incorporate the graft. This failed incorporation has been well documented histologically as fibrous pseudomembrane lining the seroma wall and immature fibroblasts lining the graft [11,13]. When evaluating this complication, differential diagnosis should include injury of the minor lymphatic duct or its branches, resulting in local lymph collection (the so called lymphocele) [1]. In fact, Strauch et al reported lymphocele in 5 patients, with 2 of these patients requiring aspiration [1]. Lymph is easily recognized after aspiration, because of its characteristic milky color, while biochemical analysis reveals the presence of chylomicrons, high triglyceride levels and cholesterol/triglycerides ratio <1 [14]. In our patient, the diagnosis of lymphocele was excluded because aspirated fluid did not have any of the above characteristics. This is the first reported case of a seroma following axillary artery repair with a graft, after arterial cannulation for CPB. Interestingly, seroma in our case was induced by a small polytetrafluoroethylene (PTFE) patch, indicating the possible qualitative (rather than quantitative) role of the synthetic graft. In our opinion, low postoperative hematocrit, decreased plasma oncotic pressure, hypertension, and presence of fat-rich subcutaneous tissue in the axillary perigraft space were factors promoting seroma formation in our patient. Indeed, Dauria et al [11] claimed that a decrease in hematocrit by one-half resulted in three-fold increase of graft weeping in renal patients undergoing arterio-venous graft placement. Management options for persistent seromas include conservative, interventional and surgical therapies. Conservative management consists of repeated aspiration, topical application of microfibrillar collagen or histoacryl tissue, wrapping with collagen fleece soaked in fibrin glue or absorbable collagen, intraluminal injection of hemostatic fibrin glue, plasmapheresis (10-12 sessions), or stent implantation [15-17]. However, repeated aspiration increases graft infection risk to 12% [18] and should be performed with strict sterile precautions. It is worth noting that, compared to other seroma locations, external local compression by gauze package has less beneficial effect in the subclavian area due to deep location of the cannulation site. Injection of a sclerosing agent can result in later graft thombosis [16] and is not recommended. However, case reports of microfibrillar collagen (the end-product of mature fibroblasts) insertion into the space surrounding an axillo-bifemoral graft have documented successful graft incorporation into the surrounding tissue without fluid re-accumulation [16]. Surgical seroma treatment is only indicated when conservative management has failed, the recurring fluid collection is > 2 cm in diameter, there is impending skin necrosis, or the graft is infected [11,18,19]. In such cases, surgical treatment consists of excision of the sac and replacement of the graft using a new synthetic graft or an umbilical vein or homograft iliac artery [17,19]. Conservative management is successful in only 65-70% of cases, due to high rates of recurrence and infection [16]. In contrast, surgical management with replacement of the graft and radical excision of the sac has a cure rate over 92% [11,18,19].

## Consent

Written informed consent was obtained from the patient for publication of this case report and accompanying images. A copy of the written consent is available for review by the Editor-in-Chief of this journal.

## Competing interests

The authors declare that they have no competing interests.

## Authors' contributions

EA performed the operation, wrote the initial manuscript and revised the study. NB participated in the operation, collected the images, submitted and revised the manuscript. KK provided the CT scan images. MK revised and corrected the manuscript while he participated in its design and coordination. All authors read and approved the final manuscript.
